# ArabiCCR: A commercial Arabic ruling court cases dataset with judicial decisions

**DOI:** 10.1016/j.dib.2026.112844

**Published:** 2026-05-14

**Authors:** Talal Alharbi, Tuwailaa Alshammari, Nadiyah Almutairi, Mohammed Alahmadi

**Affiliations:** a Department of Information and Computer Science, College of Computer Science and Engineering, University of Ha’il, Ha’il 81481, Saudi Arabia; b Department of Science and Technology, Khafji University College, University of Hafr Al Batin, P.O. Box: 1803, Hafr Al Batin 31991, Saudi Arabia; c Department of Cybersecurity, College of Computer Science and Engineering, University of Jeddah, Jeddah 21493, Saudi Arabia

**Keywords:** Arabic Ruling cases, Data analysis, Web scraping, Natural language processing (NLP)

## Abstract

This paper presents a dataset of commercial court rulings in Saudi Arabia collected from the official Saudi Ministry of Justice website and recently made publicly available. The dataset consists of judicial decisions written in Arabic, including case narratives, legal reasoning, and final rulings. Although the original corpus includes multiple case types, this work focuses specifically on commercial cases which represent the majority of the data.

The dataset was systematically extracted, cleaned, and anonymized by the redaction of personal names to support ethical use and reproducible analysis. It provides a valuable resource for research in Arabic legal text processing and judicial reasoning. The dataset can support a wide range of applications in natural language processing and artificial intelligence, including text classification, information extraction, judgment ruling prediction, and predictive modeling. The dataset can also be used to enable comparative studies between traditional machine learning and deep learning approaches. The dataset is publicly available at: https://data.mendeley.com/datasets/np538c95yy/2.

Specifications Table

**Table:** 

Subject	Computer Sciences
Specific subject area	Deep learning, Machine learning, Text Analyses, Court Ruling Cases, Natural Language Processing (NLP).
Type of data	Table Raw, Analyzed, Filtered Data.
Data collection	The data was collected from the official Saudi Ministry of Justice website [1] by scraping means. This is achieved by using Python language, BeautifulSoup [2], Requests [3], selenium [4] packages, and stanzaNLP model [5] to redact people names.
Data source location	The data collected from https://laws.moj.gov.sa/ar/JudicialDecisionsList/1.
Data accessibility	Repository name: Mendeley Data, Zenodo Data identification number: 10.17632/np538c95yy.2 https://doi.org/10.5281/zenodo.18451654 Direct URL to data: https://data.mendeley.com/datasets/np538c95yy/2, https://zenodo.org/records/18451654
Related research article	None.

## 1 Value of the Data

• The dataset contains about 12,806 commercial cases in Arabic which have been scraped into tabular format.
• The scraped data contains judgment number, case number, court name, case type, judgment date, year, city, URL, and the full text of the case.
• The full text of the case contains sections: The Delegation (), Reasoning(), and the Ruling ().
• These sections have been extracted and separated into new columns.
• People names appearing either as Judges names, parties’ names, or names attached to corporate names have been redacted.
• The dataset offers opportunities for empirical research on judicial patterns and legal analytics in Saudi commercial law.
• The dataset facilitates information extraction and the structuring of legal knowledge from Arabic legal texts
• Enables research on automated judgment prediction and legal decision modeling using real-world judicial data.

## 2 Background

The motivation for compiling this dataset is to support planned research on the application of deep learning methods for decision prediction in the context of Saudi commercial courts. While judicial decisions are published online on the Saudi Ministry of Justice portal [1], they are not readily available in a format suitable for computational analysis. Accordingly, we collected, extracted, and structured the commercial court decisions to create a research-ready dataset. The collected corpus comprised a large number of judicial decisions, the majority of which were commercial cases. This provided an opportunity to construct a domain-specific dataset by systematically filtering, organizing, and documenting commercial court rulings.

The dataset was generated to enable empirical analysis of court decisions and to serve as input for machine learning, deep learning, and natural language processing models. The data was collected by scraping publicly accessible judgments from the Ministry of Justice judicial decisions portal and subsequently organized into structured fields separating metadata and core legal text components.

The dataset was filtered to include only commercial court cases in order to maintain legal and procedural consistency across records. This data article adds value by making the dataset accessible in a structured and reusable form. It enables transparency, reproducibility, and independent benchmarking for future studies on judgment prediction and explainable artificial intelligence in legal decision-making.

## 3 Data Description

**Fig. 1:**
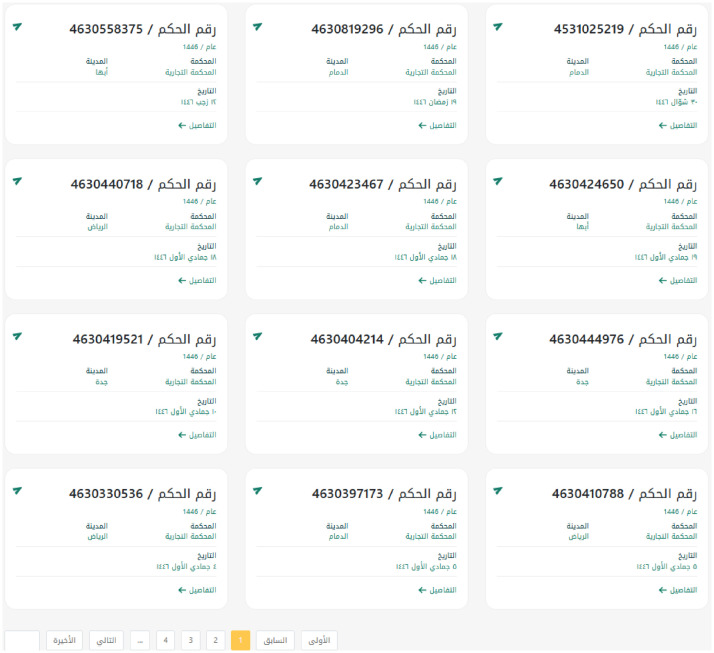
Sample of ruling list summary from the portal.

The extracted data consist of judicial decisions collected from the official Saudi Ministry of Justice portal through a structured scraping process. Each page on the portal provides a summary of 12 decisions, as shown in Fig. 1. Each case can be accessed individually by clicking on “Details” (), which directs the user to a dedicated webpage containing the case metadata and the full judgment text. The data were collected directly from publicly available judicial decisions published on the portal.

The scraped dataset initially comprised 14,857 cases. However, the objective of this study was to focus exclusively on commercial cases. Therefore, the data were cleaned and filtered to retain only cases classified as commercial, resulting in a final dataset of 12,806 cases.

The extracted data includes the case identifier, court type, decision date, case category, summary text, and other relevant metadata available on the portal. The dataset is structured in tabular format to enable systematic filtering and analysis, with each row representing a single judicial decision (i.e., one case record). In some records, the extracted text included appeal-related content, which was removed to ensure that the dataset reflects only the first-instance judgment and its associated fields.

Table 1 provides a comprehensive description of all dataset variables, along with an example value for each*.*

**Table 1:** Scraped dataset field contents by column “header”.

## 4 Experimental Design, Materials and Methods

**Fig. 2:**
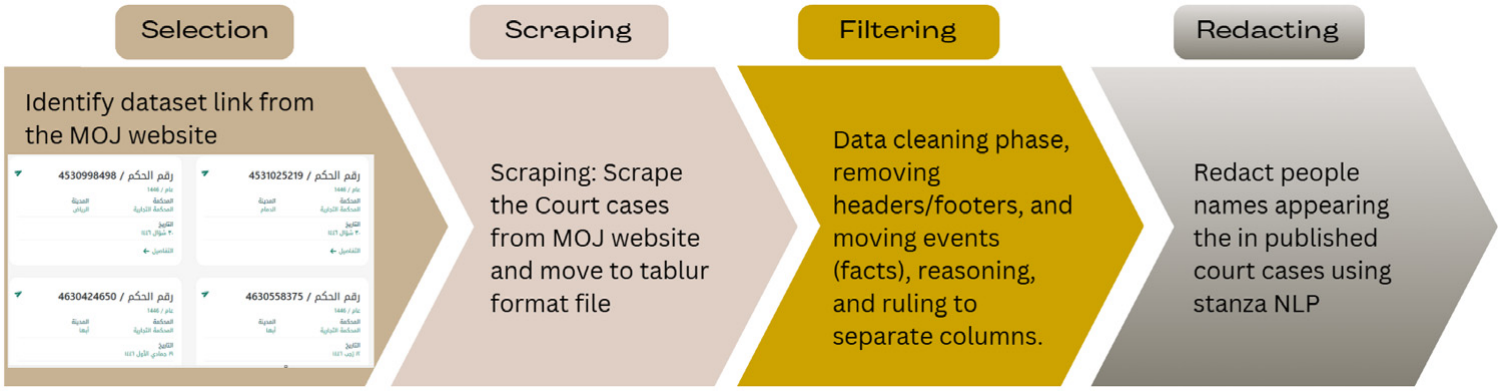
Steps taken in conducting the experiment.

The work went through several steps, as shown in Fig. 2, starting with scraping, cleaning, filtering, and redacting names. The data extraction process began by scraping all available rulings from the portal. The website displays a list of cases with metadata such as judgment number, case number, court name, city, and judgment date in Hijri format. It shows exactly 12 cases per page, and each case can be clicked to view the full judgment text.

**Fig. 3:**
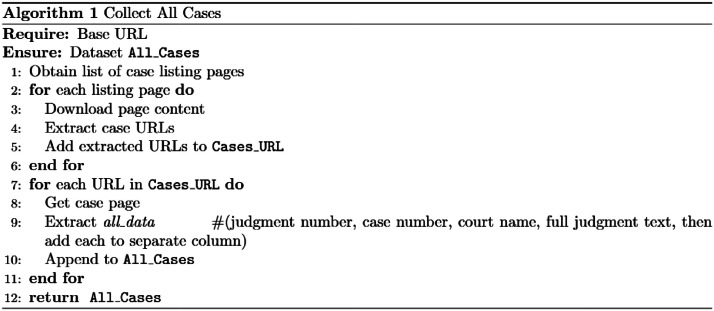
Website Scraping algorithm to collect all ruling cases.

The scraping process iterates through all pages on the website; for each page, it extracts the case metadata and then loads the full judgment text. Fig. 3 presents the pseudo-algorithm for the scraping process.

The data were collected in a tabular format (Excel file). The extraction process retrieved all cases along with header and footer content, which was subsequently removed from the judgment text. The dataset was then filtered to remove all cases that were not related to the commercial court. This was done because the aim was to prepare a commercial court dataset for future analysis and evaluation. Since most of the cases were commercial, the final dataset consisted of 12,806 cases.

When examining the judgment text, two observations were made. First, some rulings included appended appeal decisions. This occurs when one of the parties appeals the issued judgment, and the appeal information is added to the original case text. Second, each case typically contains three main sections:  (events/facts/background of the dispute),  (the court’s legal reasoning), and  (the final ruling).

**Fig. 4:**
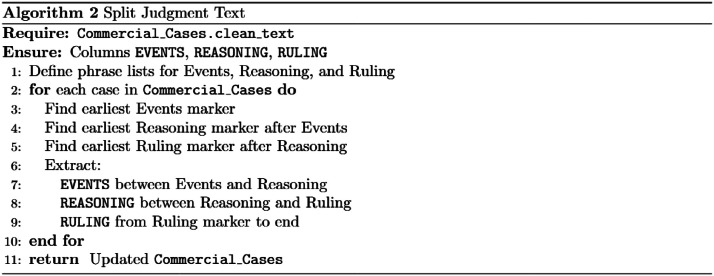
Copying text to next columns.

To maintain consistency in the dataset, all appended appeal content was removed, and only the initial ruling or verdict was retained. In addition, the *Events, Reasoning*, and *Ruling* sections were copied into separate corresponding columns while preserving the original full judgment text in its own column as depicted by algorithm 2 in Fig. 4.

The extraction of these sections was challenging because different courts, and sometimes courts in different cities, use varying wording to mark transitions between sections in the judgment text. For example, when identifying the beginning of the events section, the leading term varied across rulings, including: ["", "", "()", "()", "()", "()"]. The complete list of section identifiers is presented in Table 2.

**Table 2:** Keywords before each section.

During text examination, we found that some names were retained and not redacted. Therefore, the final step was to redact all people names (appearing in the last four columns the full *judgment tex*t, *events, reasoning*, and *ruling* columns) by replacing them with [Person Name], see Fig. 6. We used the stanza NLP tool to identify these names.

To improve accuracy, the redaction process was performed in two steps. First, we identified specific leading words after which names commonly appear, leading terms include “” (Plaintiff), "" (Defendant), "" (Accused), "" (Appellant), “” (Respondent in appeal), "" (the person named), "" (Mr.), "" (Ms./Mrs.), "" (Representative/Agent), "" (Lawyer/Attorney), "" (Judge), "" (Company), "/" (Defendant/), "/" (his client/), and "" (for the plaintiff).

**Fig. 5:**
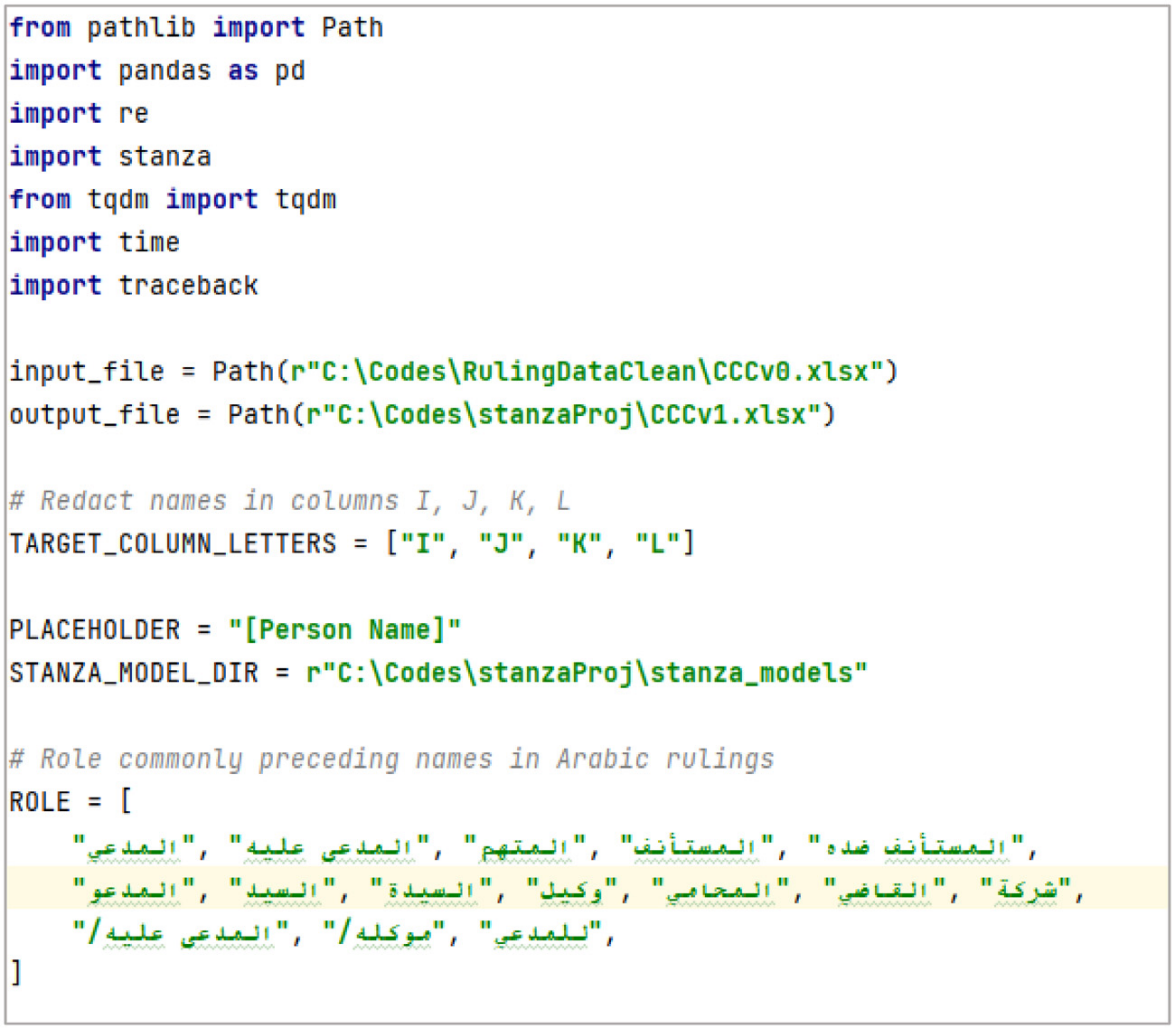
Snippet of name redaction using Stanza.

The second step involved identifying tokens labeled as PERSON entities by the NLP model and redacting them accordingly. This was implemented using the Stanza Arabic NER model with default settings, where all detected names were replaced with a standardized placeholder (“[PERSON NAME]”). Fig. 5 presents a snippet of the redaction code, while the complete implementation is available in [6].

**Fig. 6:**
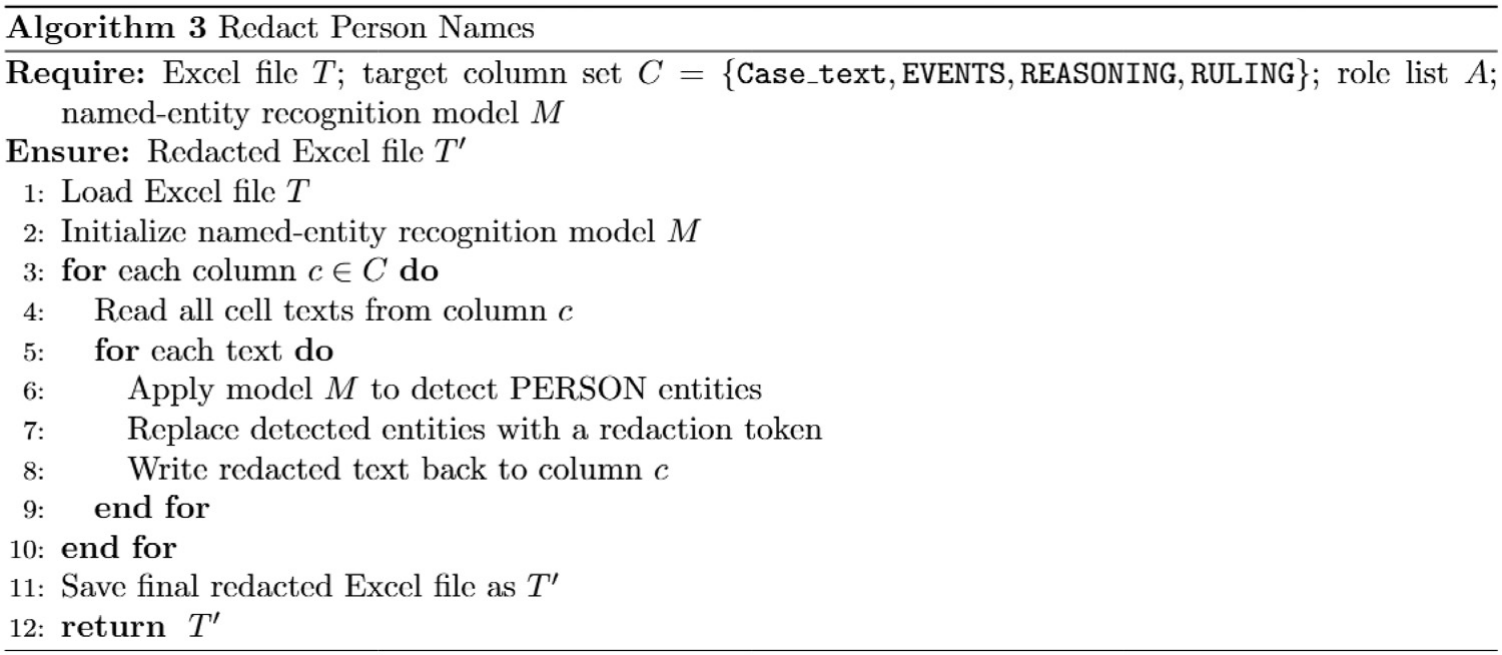
People names redaction algorithms.

**Fig. 7:**
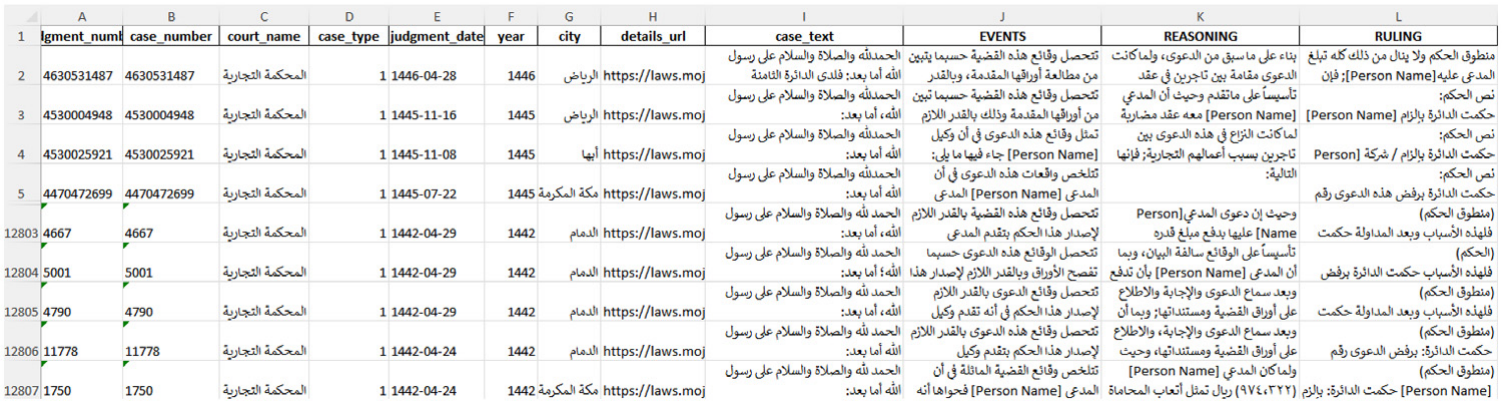
Dataset final format.

The produced dataset comprises 12,806 cases, where each column contains either metadata or judgment text. The full judgment text is provided in column I, and copies of the events, reasoning, and ruling are presented in columns J, K, and L, respectively as shown in Fig. 7. A clear header list for the dataset with description is presented in Table 3.

**Table 3:** All field contents by column “header”.

Column	Title	Description
A1	judgment_number	Judicial ruling number by the court.
B1	case_number	Case number registered in the court system.
C1	court_name	Court issued the judgment (Commercial Court).
D1	case_type	Classification of the case according to its legal domain (e.g., commercial, civil, labor).
E1	judgment_date	Date on which the final judgment was issued by the court.
F1	year	Year when the judgment was issued.
G1	city	City where the court is located.
H1	details_url	URL linking to the official online source.
I1	case_text	Full case text.
J1	EVENTS	Section of the judgment describing the background and events of the case.
K1	REASONING	Legal reasoning, analysis, and justification for the decision.
L1	RULING	Final judgment decision.

## Limitations

This dataset was collected from rulings that were publicly available on the official Saudi Ministry of Justice website, which may result in certain limitations. Firstly, it only includes only the cases that were available on the website during the data collection period. Secondly, the dataset focuses on commercial cases in Saudi Arabia and it reflects a specific legal context that may require adaptation when applied to other legal domains or countries.

## Ethics Statement

The authors have read and followed the ethical requirements for publication in Data in Brief and confirm that the current work does not involve human subjects, animal experiments, or any data collected from social media platforms.

## CRediT Author Statement

**Talal Alharbi:** Conceptualization, Methodology, Investigation, Software; **Tuwailaa Alshammari:** Methodology, Investigation, Software, Writing - original draft; **Nadiyah Almutairi:** Conceptualization, Methodology, Writing, Reviewing; **Mohammed Alahmadi:** Methodology, Validation, Supervision, Review & Editing.

## Data Availability

Mendeley DataArabiCCR Dataset (Original data).
